# Using BAC transgenesis in zebrafish to identify regulatory sequences of the amyloid precursor protein gene in humans

**DOI:** 10.1186/1471-2164-13-451

**Published:** 2012-09-04

**Authors:** Leighcraft A Shakes, Hansen Du, Hope M Wolf, Charles Hatcher, Derek C Norford, Patricia Precht, Ranjan Sen, Pradeep K Chatterjee

**Affiliations:** 1 Julius L. Chambers Biomedical/ Biotechnology Research Institute & Department of Chemistry, North Carolina Central University, 1801 Fayetteville Street, Durham, NC 27707, USA; 2 Laboratory of Molecular Biology and Immunology, National Institute on Aging, Biomedical Research Center, 251 Bay View Blvd., Suite 100, Baltimore, MD, 21224, USA; 3 Present address: Department of Chemistry, University of North Carolina at Chapel-Hill, Chapel-Hill, NC, 27599, USA

## Abstract

**Background:**

Non-coding DNA in and around the human Amyloid Precursor Protein (APP) gene that is central to Alzheimer’s disease (AD) shares little sequence similarity with that of *appb* in zebrafish. Identifying DNA domains regulating expression of the gene in such situations becomes a challenge. Taking advantage of the zebrafish system that allows rapid functional analyses of gene regulatory sequences, we previously showed that two discontinuous DNA domains in zebrafish *appb* are important for expression of the gene in neurons: an enhancer in intron 1 and sequences 28–31 kb upstream of the gene. Here we identify the putative transcription factor binding sites responsible for this distal *cis*-acting regulation, and use that information to identify a regulatory region of the human APP gene.

**Results:**

Functional analyses of intron 1 enhancer mutations in enhancer-trap BACs expressed as transgenes in zebrafish identified putative binding sites of two known transcription factor proteins, E4BP4/ NFIL3 and Forkhead, to be required for expression of *appb*. A cluster of three E4BP4 sites at −31 kb is also shown to be essential for neuron-specific expression, suggesting that the dependence of expression on upstream sequences is mediated by these E4BP4 sites. E4BP4/ NFIL3 and XFD1 sites in the intron enhancer and E4BP4/ NFIL3 sites at −31 kb specifically and efficiently bind the corresponding zebrafish proteins *in vitro*. These sites are statistically over-represented in both the zebrafish *appb* and the human APP genes, although their locations are different. Remarkably, a cluster of four E4BP4 sites in intron 4 of human APP exists in actively transcribing chromatin in a human neuroblastoma cell-line, SHSY5Y, expressing APP as shown using chromatin immunoprecipitation (ChIP) experiments. Thus although the two genes share little sequence conservation, they appear to share the same regulatory logic and are regulated by a similar set of transcription factors.

**Conclusion:**

The results suggest that the clock-regulated and immune system modulator transcription factor E4BP4/ NFIL3 likely regulates the expression of both *appb* in zebrafish and APP in humans. It suggests potential human APP gene regulatory pathways, not on the basis of comparing DNA primary sequences with zebrafish *appb* but on the model of conservation of transcription factors.

## Background

It is important to understand the regulation of the Amyloid Precursor Protein (APP) gene expression because epidemiologic studies show that Alzheimer Disease (AD) is exquisitely sensitive to gene dosage [1], and levels of APP expression including β-peptide levels correlate with the severity and age-of-onset of AD [2]. The severity and onset of AD is thus closely linked to expression of the APP gene. These observations suggest that controlling APP gene expression is a possible route to reducing the severity of AD. A pre-requisite for therapeutic manipulation of APP gene expression is a more complete understanding of the mechanisms that regulate APP expression in neurons. The APP gene promoter does not contain a functional TATA box but instead has long CpG islands and a strong initiator element (INR) surrounding the major transcription start site [3]. While transcriptional regulation of APP gene has been studied extensively, most of that work has focused on the proximal ~ 1500 bp sequences of the promoter [3-13], and it is unclear to what extent APP gene is regulated by promoter sequences alone. Like most other genes it is likely that the APP promoter is modulated by distal regulatory sequences. The non-coding DNA within and surrounding the APP gene is not conserved in vertebrates, and although ~700 bp of DNA immediately upstream of the start site is conserved in mammals, this conservation does not extend to other vertebrates such as Fugu or zebrafish [3,14]. Thus regulation of the gene by *cis*-acting distal sequences remains poorly understood. Although regulatory function can be conserved across species without sequence similarity [15-19], identifying such sequences that control gene expression under those circumstances is much more difficult.

We have previously shown that two discontinuous DNA regions regulate neuron-specific *appb* gene expression in zebrafish. One of these is an enhancer located within intron 1; in the absence of this enhancer there is no expression of a BAC transgene that contained approximately 100 kb of 5’ sequences [14]. The second regulatory sequence mapped to a region located between approximately 28–31 kb 5’ of the transcription start site of the zebrafish *appb* gene. Deletion of this element shifted the expression pattern from being neuron-specific to notochord-specific, which is the default pattern observed with the basal promoter plus intron-enhancer combination. Based on these observations, we proposed that the upstream element suppressed aberrant expression (in the notochord) and activated appropriate expression in neurons. Requirement of the upstream-enhancer for expression further suggested that zebrafish *appb* is regulated by interaction between these distal regulatory sequences.

Here we identify the putative transcription factor binding sites that mediate activity of these regulatory regions and use the information to study the regulation of the human APP locus. Analysis of the expression of enhancer-trap BACs containing mutated intron 1 enhancers in zebrafish indicates that binding sites of at least two known transcription factors are important for function. They are the clock-regulated and immune system modulator transcription factor E4BP4/ NFIL3 and members of the Forkhead gene family (XFD1). A search of non-coding DNA in introns and the 50 kb sequence surrounding the *appb* gene for additional binding sites revealed a ~ 8-fold and ~ 11-fold greater than statistical frequency of E4BP4 and XFD1 sites, respectively. Amongst these is a cluster of three E4BP4 sites at −31 kb. These sites bound the E4BP4 DNA binding domains, expressed in *E. coli,* efficiently and selectively *in vitro*. Though comparison of zebrafish and human APP did not reveal substantially conserved non-coding sequences that could represent regulatory elements, we hypothesized that gene expression may be conserved via the use of the same transcription factors. Therefore, we searched for E4BP4 binding sites in the human APP locus. Remarkably, we found that putative E4BP4 sites were also over-represented in the human APP locus, though their locations differed from that seen in the zebrafish *appb*. One such cluster of four E4BP4 binding sites in the fourth intron of the human APP gene was marked by a peak of acetylated histones in a human neuroblastoma cell line that expresses APP. We propose that E4BP4/ NFIL3 may regulate human APP expression via binding to distal regulatory sequences.

## Methods

### BAC clones

BACs CH211-192O20 and CH211-43O16 from a zebrafish library, designated here as BACs C and D respectively, have been described [14]. The two BACs overlap one another and contain different lengths of sequences upstream of *appb* gene. Both were used in order to have maximum upstream DNA, both closest to and farthest from the *appb* transcription start site, in the enhancer-trap BACs. This was necessitated by the ~110 kb packaging capacity of the phage P1 head used in the generation of enhancer-trap BACs (see Figure 7 of reference [14]).

### Generating enhancer-trap BACs

Progressive truncations from either end of BAC DNA, purification and analyses of clone DNA from deletion libraries using Field Inversion Gel Electrophoresis (FIGE) was performed using procedures described before [20-22]. Sequence of the newly created end was determined in each case using primers from the Tn10 transposon end remaining after the truncation.

### BAC DNA injections into zebrafish eggs

Injections of Qiagen tip-purified enhancer-trap BAC DNA into zebrafish eggs and subsequent analyses of EGFP expression in developing embryos using fluorescence microscopy were performed as reported earlier [14]. To generate transgenic lines, enhancer-trap BAC DNA with iTol2-end insertions were co-injected with Tol2 transposase mRNA as described previously [23].

### Mutagenesis of intron 1 enhancer in Enhancer-trap transposon plasmid

Suitable PCR primers were used to amplify segments of the 1 kb intron enhancer, and the amplified products incorporated into the enhancer-trap Tn10 transposon plasmid. Point mutations were engineered into PCR primers so that the amplified product contained mutations in putative transcription factor binding sites that overlapped such as SOX5 and E4BP4. Thus to get mutations in only SOX5 and not E4BP4, point mutations were introduced. Changes to the putative binding sites were first incorporated into the small plasmid containing the enhancer-trap Tn10 which was then inserted into *appb* BACs C and D, exactly as described previously to make enhancer-trap BACs with the wild type sequence of the intron enhancer [14].

### Preparing zebrafish Forkhead and E4BP4/ NFIL3 proteins

Conserved DNA binding domains (DBD) of the zebrafish Forkhead and E4BP4 genes were amplified from zebrafish genomic DNA using primers shown in Additional file 1: Figure S1. Restriction sites were introduced in-frame to the Forkhead and E4BP4 open reading frames (ORFs) to facilitate cloning into the pET-30a(+) expression vector. A six-histidine residue tag fused to the N-terminal end of these proteins was used for purification purposes as previously reported [24]. DBD of Forkhead and E4BP4 were purified from bacteria as previously described [25]. Electrophoretic Mobility Shift Assays (EMSA) were performed exactly as described earlier [25].

### Chromatin Immunoprecipitation with H3K9Ac antibody

ChIPs, real-time PCR, and data analysis were performed as described [26]. The anti-H3K9Ac antibody was purchased from Abcam, Cambridge, MA. The control antibody anti-IgG was obtained from Millipore, Billerica, MA. Human neuroblastoma SHSY5Y cells were propagated in an undifferentiated state, cultured in DMEM medium and 10% heat inactivated FBS. H3K9Ac ChIP was performed on undifferentiated SHSY5Y cells. ChIP primers were designed to span potential E4BP4 binding sites, and are displayed in Additional file 2: Figure S2. Primers used for detecting mRNA levels of E4BP4 in the undifferentiated cell line SHSY5Y are displayed in Additional file 3: Figure S3.

### Identification of putative transcription factor (TF) binding sites

The sequence in the 1 kb intron 1 enhancer of *appb* was analyzed using the “MotifScanner” program, and the results are shown in Additional file 2: Figure S2 of reference [14]. The putative TF binding sites with the highest probability scores from that analysis are highlighted in the intron enhancer sequence shown here in Additional file 4: Figure S4. Mutational analyses of putative TF binding sites within the intron 1 enhancer revealed that E4BP4/ NFIL3 and XFD1 sites were required for function. Next, the genomic DNA sequence containing either the zebrafish *appb* gene or the human APP gene, displayed in a Microsoft Word file, were scanned by the “Find” function for the sequence representing the binding sites of E4BP4/ NFIL3 or XFD1. The reverse strand binding sites for the two transcription factors were similarly identified using the “Find” function on the sequences complementary to the sites. The total of these constituted the putative binding sites of each transcription factor.

### Sequence motif frequencies

Frequencies for random occurrence of putative binding sites were calculated by raising ¼ (which represents the probability of finding a specific nucleotide at any location) to the total number of nucleotides in the consensus binding site for the specific transcription factor. The fold over-represented was deduced from the ratio of actual occurrence at the genetic locus to what would be expected if they occurred randomly.

## Results

**Figure 1:**
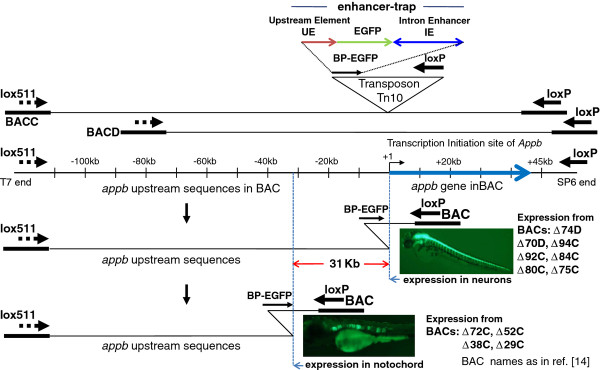
**Scanning the *appb *genomic region of zebrafish by enhancer trapping using BACs represented schematically (summary of results from reference**[14]**). ** The two BACs C and D used in this study overlap one another and contain different lengths of sequences upstream of *appb* gene. They are shown schematically as the top two lines. The inverted triangle represents enhancer-trap in Tn10 transposon, which is comprised of 0.35 kb of DNA immediately upstream of *appb* (UE), followed by EGFP gene with basal promoter, and ~1 kb intron 1 enhancer (IE). The entire enhancer-trap cassette is named BP-EGFP. The *appb* gene region in the BACs, with the thick blue arrow to represent the total length of exons and introns of the gene, is drawn to scale and shown below it. Insertion of the enhancer-trap into the *appb* BAC DNA and subsequent Cre recombination between the transposed loxP and the BAC-end loxP deletes the BAC DNA from that end and simultaneously inserts the enhancer-trap (shown as BP-EGFP). The enhancer-trap is in front of loxP in the transposon and is retained in the BAC after Cre-mediated loxP-loxP deletion. This end-truncation is represented by the bent line to illustrate the location of that transposon-end retained in the BAC after the loxP-Cre deletion. The earlier study [14] found that *appb* BACs that had the enhancer-trap located close to the *appb* transcription start site expressed EGFP fluorescence in neurons (e.g. BACs Δ74D, Δ70D, Δ94C, Δ92C Δ84C, Δ80C, Δ75C), while *appb* BACs that had the enhancer-trap inserted further upstream beyond -31 kb of the *appb* gene expressed EGFP fluorescence in the notochord (e.g. BACs Δ72C, Δ52C, Δ38C, Δ29C). The vertical blue dotted lines, separated by ~31 kb, mark these locations on the *appb* BACs. The names of BACs are indicated adjacent to the pictures of EGFP expression in zebrafish neurons or the notochord.

New technology for scanning the zebrafish *appb* gene locus with enhancer traps using BACs was reported in an earlier study [14], and is illustrated in Figure 1. The enhancer-trap comprised of a basal promoter EGFP gene flanked by 0.35 kb of sequence immediately upstream of the *appb* transcription initiation site and ~ 1 kb of DNA containing the intron 1 enhancer of *appb* (shown schematically in top panel of Figure 1). The enhancer-trap is located in front of the loxP arrowhead in the Tn10 transposon that is inserted randomly into BACs C and D overlapping the *appb* locus. Cre-mediated recombination between this inserted enhancer-trap-loxP and the loxP endogenous to the BAC generates libraries of *appb-*BAC deletions in which the loxP end is brought progressively closer to the lox511 end; while simultaneously placing the enhancer-trap at the new loxP end. The set of enhancer-trap *appb-*BACs is first characterized by sequencing the new ends and then expressed in zebrafish embryos. Both BACs were needed for maximal representation of upstream sequences both proximal and distal to *appb* transcription start site in enhancer-trap BACs (see Methods, and reference [14]).

Results from that previous study indicated that the enhancer in intron 1 can function specifically in non-neural tissue such as the notochord, where endogenous *appb* is not expressed, when used with promoter proximal elements within the +0.147 to −0.35 kb of sequence surrounding the transcription start site of *appb*. Thus expression in notochord was observed using either the small enhancer-trap transposon plasmids containing only the proximal promoter elements, or enhancer-trap *appb-*BACs in which sequences −0.35 to −31 kb had been deleted. However when additional 5’ sequences extending till approximately −31 kb in the enhancer-trap *appb-*BACs were present, gene expression became exquisitely specific to neurons (the vertical dashed blue lines in Figure 1 demarcate the end-points of deletions in the enhancer-trap BACs that produce the two distinct expression patterns). These results suggest that the intron enhancer works with sequences in the −31 kb region to confer neuron-specific gene expression. The results also appeared to suggest that some type of transcription repression occurs by factors binding to sequences between −28 and −31 kb to suppress expression in the notochord, as deduced from the different expression patterns of BACs Δ75C and Δ72C [14]. Simultaneous expression in the notochord and neural cells was not observed in the same fish using BAC deletions Δ74D through Δ75C (Summarized here in Figure 1 from the data shown in Figure 7, Panels A-C of reference [14]). We concluded that tissue-specific expression of the *appb* gene resembling its endogenous pattern required two separate, somewhat distant regulatory domains to cooperate in *cis* to confer tissue-specificity. The two domains of regulation are the ~ 1 kb of DNA within intron 1 (ZFISH7:9:29144733–29145740), and the region +0.147 to −31 kb. As indicated in the earlier study, exclusion of the ~1 kb intron element produced no expression of GFP: *appb* BACs with ~100 kb of 5’ sequences without the intron 1 enhancer, i.e. BACs deleted from the wild type loxP end of insert DNA with Tn-US that lacked the intron enhancer (Figures 1B, 1C, &2 in reference [14]) failed to express GFP in any tissue. Absence of the region −0.35 to −31 kb on the other hand led to expression in the inappropriate tissue, the notochord, as seen with BACs Δ72C, Δ52C, Δ38C, and Δ29C.

**Figure 2:**
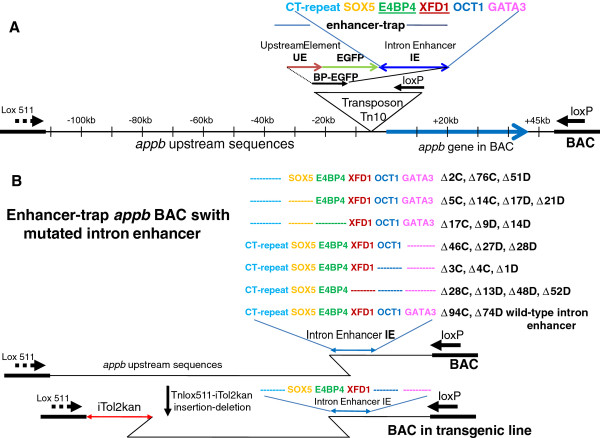
**Panel A: Schematic representation of bio-informatically predicted transcription factor binding sites in the intron 1 enhancer (IE) within the enhancer-trap (taken from Additional file **2**: Figure S2 of reference **[14]**), highlighted by the colored letters. Panel B:** Mutated enhancer-trap BACs used in this study: Mutations and deletions, represented schematically with dotted lines of same color in place of the letters, were engineered into the intron enhancer (IE) in the small enhancer-trap transposon plasmid at the sites marked by **CT-repeat, SOX5, E4BP4, XFD1, OCT1, and GATA3.** Each mutated enhancer-trap transposon was inserted into *appb* BACs C and D to generate the enhancer-trap BACs indicated on the side of the corresponding mutation. For example the blue dashed line in the first row indicates a deletion of the CT-repeat sequence in enhancer-trap (shown as dotted blue line), and the BACs containing this mutation are Δ2C, Δ76C and Δ51D. Because SOX5 and E4BP4 sites overlapped, only point mutations were introduced into the SOX5 site to obtain the plasmid with wild type E4BP4 and point mutation in SOX5 and deletion of CT-repeat, shown in second row Panel B. Enhancer-trap BACs with these mutations are Δ5C Δ14C, Δ17D Δ21D. Row 7 shows the wild type enhancer-trap with Δ94C, and Δ74D as representatives. The results of expressions of these BACs are summarized in Table 1. The last enhancer-trap BAC in Panel B (row 8) has enhancer-trap deleted for CT-repeat, OCT1 and GATA3. This BAC is also deleted from the lox511 end of BAC with the Tnlox511-iTol2kan to make the germline transgenic zebrafish shown in Figure 3, Panel E. The bent lines on both ends of the BAC represent end-truncations by the transposons, and illustrate the location of the particular transposon end preserved after either the loxP-loxP or lox511-lox511 deletions mediated by Cre protein, respectively.

### Expression analyses of *appb*-BACs with mutated intron 1 enhancer: intact putative E4BP4 and XFD1 sites essential for *appb* expression in zebrafish

**Figure 3:**
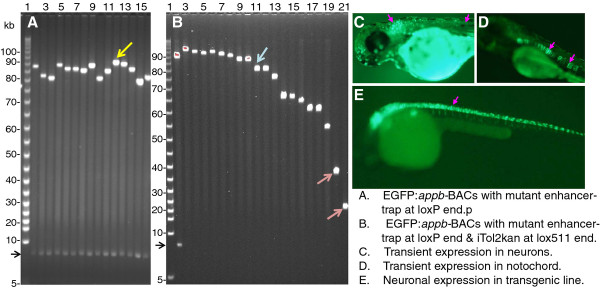
**Panel A: FIGE analysis of enhancer-trap BACs with mutated intron 1 enhancer from different libraries. Panel B:** FIGE analysis of clone DNA from the library generated by inserting Tnlox511-iTol2kan at the lox511 end of the enhancer-trap BAC in lane 12, Panel A (marked by yellow arrowhead). **Panel C:** EGFP fluorescence from transient expression in neurons (marked by the pink arrowheads) of zebrafish injected with mutated but functional intron enhancer-trap BAC with intact upstream DNA, **Panel D:** EGFP expression in notochord (indicated with pink arrowheads) from injecting enhancer trap BAC with mutated but functional intron enhancer and with 31 kb upstream DNA deleted (such as clone in lane 21, panel B, red arrowhead) taken from the same library as the BAC used for Panel C. **Panel E:** EGFP fluorescence in neurons (marked by the pink arrowhead) from a F2 transgenic zebrafish line obtained from the enhancer-trap BAC shown in lane 11 of Panel B (marked by blue arrowhead). The mutated but functional intron enhancer used was deleted for GATA3, OCT1 and the CT-repeat element (clone shown schematically in row 8, Panel B of Figure 2). Additional examples of germline transgenic fish with slightly smaller enhancer-trap BAC transgenes, but containing the upstream ~31 kb sequence, are shown in Figure 6 panels A and B of reference [23]. The BAC vector DNA band from Not I digestion is shown by the black arrowhead to the left of panels A and B. Lane 2, Panel B, contains the same DNA as lane 12, Panel A. Lanes 3–21 in panel B do not have this band because insertion of Tnlox511-iTol2kan at the lox511 end of BAC DNA and subsequent lox511-lox511 deletion eliminates the Not I site at that end [23].

Our previous study also reported a bio-informatic analysis of putative transcription factor binding sites within the intron 1 enhancer sequence (Additional file 2: Figure S2 in reference [14]). Sites with the highest probability scores in that list, such as SOX5, E4BP4, XFD1, OCT1 and GATA3, are shown schematically here in the intron enhancer (IE) of the enhancer-trap transposon in Figure 2A. Deletions from either end of the intron enhancer sequence and point mutations, to distinguish between overlapping sites, were made in these putative transcription factor binding sites in the 1 kb intron enhancer of *appb*. Changes to the binding sites were first incorporated into the small plasmid containing the enhancer-trap Tn10 transposon (shown as the inverted triangle in Figure 2A) which was then introduced into *appb* BACs C and D, exactly as described previously to make enhancer-trap BACs with the wild type sequence of the intron enhancer. A schematic representation of all enhancer-trap BACs containing mutated intron enhancers that were used in this study is shown in Figure 2B. Color-coded letters indicate the locations of the wild type binding sites of transcription factors in intron enhancer (IE), while the dashed colored lines represent deletions of that particular site. The identities of mutated enhancer-trap BACs from each mutant intron enhancer, used for expression in zebrafish in this study, are indicated adjacent to the changes made. Mutant enhancer-trap BACs chosen for expression had deletion-ends within −31 kb of the *appb* transcription start site. Most deletion-ends were within 10 kb, and some such as Δ28D and Δ1D had deletion-ends <2 kb of the transcription start site. The deletion-ends indicate locations of the enhancer-trap in BACs, and are represented schematically with the bent lines in Figures 1 and 2. The lowest panel in Figure 2B shows a mutated enhancer-trap BAC that was again deleted from the opposite end by a lox511-iTol2kan transposon. Enhancer-trap BAC DNAs were injected into zebrafish embryos for expression as transgenes. Locations of intron enhancer sequence-changes, and the PCR primers used to construct them, are depicted schematically in Additional file 4 Figure S4. A total of 18 mutated enhancer-trap transposon-inserted BAC libraries were made, some with a mixture of enhancer-traps containing the modified intron enhancer in both orientations. Experiments injecting DNA into zebrafish eggs were repeated at least four times with BACs that expressed EGFP fluorescence in neurons, and six times with BACs that did not express EGFP. The results obtained with transient expressions are summarized in Table 1 and indicate that the putative binding sites for OCT1, GATA3, SOX5 and the CT-repeat element are dispensable for *appb* expression in zebrafish neurons. Clones such as Δ94C or Δ74D that contain wild type intron enhancer (Figure 2B) generate identical expression patterns as Δ5C with CT-repeat and SOX5 deleted, or Δ1D with GATA3 and OCT1 deleted (Figure 2B, Table 1). In contrast, DNA sequences that contain E4BP4 or XFD1 sites are critical for expression of *appb* in neurons of zebrafish. When mutated or deleted, the resulting enhancer-trap BACs do not express either in neurons or the notochord. A FIGE of representative larger than 75 kb *appb* BACs with mutated intron 1 enhancers are shown in panel A of Figure 3. The enhancer-traps in these BACs are located well within 31 kb of the start site of transcription of *appb*.

**Table 1:** **EGFP expression patterns of mutant Enhancer-trap *appb *BACs**

**Enhancer -trap BAC injected**	**Intron enhancers**	**Average of 4 or 6 injections**		
	**Deletions from CT-repeat end**	**# of eggs injected**	**# of embryos survived**	**# of expression in neurons**
Δ94C	CT+ve	75	32	10
Δ2C, Δ76C, Δ51D	CT-ve	90	40	8
Δ5C, Δ14C, Δ17D, Δ21D	CT-ve, SOX 5-ve	110	33	9
Δ17C, Δ9D, Δ14D	CT-ve, SOX 5-ve, E4BP4-ve	140	70	0
	**Deletions from GATA3 end**			
Δ46C, Δ27D, Δ28D	GATA3-ve	105	42	12
Δ3C, Δ4C, Δ1D	GATA3-ve, OCT1-ve	150	100	8
Δ28C, Δ13D, Δ48D, Δ52D	GATA3-ve, OCT1-ve, XFD1-ve	130	55	0

Mutations were engineered into the intron 1 enhancer (IE) of the enhancer-trap and inserted into *appb* BACs C and D. Structures of mutant enhancer-trap BACs shown schematically in Panel B of Figure 2, and their expression patterns summarized here in Table 1.

A few of the BACs that expressed in neurons were further retrofitted with iTol2-ends at the opposite lox511 end of BAC DNA for germline propagation, using the transposon pTnlox511-iTol2kan as described recently [23]. For example the BAC clone in lane 12 of Panel A, Figure 3, (marked by yellow arrowhead), was truncated from the lox511 end of BAC DNA using lox511-iTol2kan transposon. Clone DNAs from the deletion/retrofitting library is shown in Panel B. Clone DNA from lane 11 in Panel B (indicated by blue arrowhead) was introduced into zebrafish eggs for germline propagation, and a F2 transgenic fish representative of this line is shown in Panel E. It has an iTol2kan-EGFP-*appb-*BAC transgene with intron 1 enhancer deleted for GATA3, OCT1 and the CT-repeat sequences (DNA of this BAC clone is shown schematically in bottom panel of Figure 2B).

### Distribution of E4BP4 and XFD1 sites in zebrafish *appb* gene

**Figure 4:**
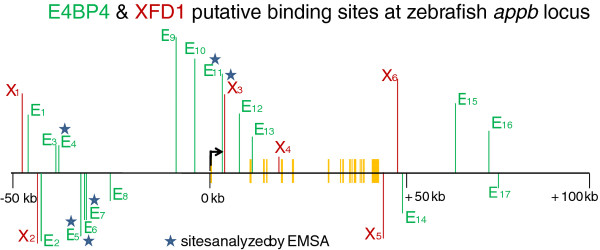
**Location of E4BP4 (E, in green), and XFD1 (X, in red), sites in non-coding DNA within zebrafish *appb *and surrounding 50 kb DNA.** The green and red vertical lines above or below the horizontal line indicate sites in the forward and reverse strand of DNA, respectively. The short yellow vertical bars indicate exons of *appb.* Stars mark the E4BP4 and XFD1 sites shown to specifically bind the DNA-binding domains of zebrafish E4BP4 or Forkhead proteins respectively, by EMSA. The bent arrow indicates transcription start site of *appb* gene.

A search of the genome database for additional E4BP4 and XFD1 sites within and 50 kb sequence surrounding the zebrafish *appb* gene revealed that both these sites are highly over-represented in the gene region, approximately 8-fold and 11-fold over statistical frequency, respectively. These are shown schematically in Figure 4. Amongst these is a cluster of three E4BP4 sites at −31 kb. Locations of these sites are also tabulated in the top panel of Additional file 5: Figure S5.

Sequence analysis of the new ends created by the lox511-iTol2kan transposon in BAC DNAs in lanes 20 and 21, marked by the red arrowheads in Panel B, Figure 3, indicated that the lox511-transposon had deleted the cluster of three E4BP4 sites at −31 kb (Figure 4). When injected into zebrafish embryos neither of these DNAs expressed EGFP in neurons. Instead, expression patterns were always in the notochord (Figure 3D), demonstrating that the cluster of three E4BP4 sites at ~31 kb upstream of *appb*, shown schematically in Figure 4, is necessary for neuron-specific expression.

### Conserved DNA binding protein domains of zebrafish Forkhead and E4BP4 specifically recognize the XFD1 and E4BP4 sites in intron enhancer and −31 kb of *appb* gene *in vitro*

**Figure 5:**
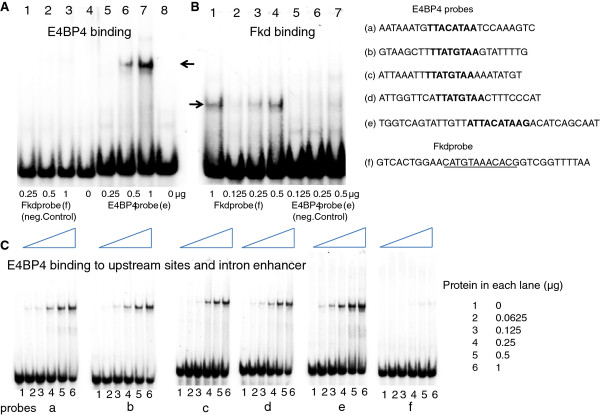
**The DNA binding domain common to members of zebrafish E4BP4 or Forkhead (Fkd) families were each expressed in *E. coli, *partially purified under non-denaturing conditions, and tested for specific binding to their recognition sequences identified in and around the zebrafish *appb *gene. Panel A:** E4BP4 protein, indicated below each lane in μg, added to Fkd probe (f), lanes 1–3, or the E4BP4 probe (e), lanes 4–8. **Panel B:** Fkd protein, indicated below each lane in μg, added to Fkd probe (f), lanes 1–4, or E4BP4 probe (e), lanes 5–7. **Panel C:** E4BP4 protein binding to probes (a through f) spanning upstream E4BP4 sites (a-d), E4BP4 site in intron 1 enhancer (e), and Fkd site in intron enhancer (f). E4BP4 protein used in each lane (μg) is indicated on right. Probes (b-d) correspond to cluster of three E4BP4 sites at −31 kb.

We next determined whether the proposed E4BP4 and XFD1 sites in these regulatory regions bound the corresponding zebrafish proteins. For this we expressed the conserved DNA binding domains (DBD) of zebrafish E4BP4 (147 aa) and Forkhead (154 aa) in *E. coli.* (shown schematically in Additional file 1: Figure S1). The DNA binding domains were partially purified under non-denaturing conditions using the histidine tag, and used in Electrophoretic Mobility Shift Assays (EMSA). As EMSA probes we used probes e and f from intron 1 of *appb* (Figure 5). Probe e, spanning site E11 in Figure 4, formed a discrete nucleoprotein complex only with recombinant E4BP4-DBD (Figure 5A lanes 5–8) indicated by the arrow, whereas probe f, spanning site X3 in Figure 4, bound only to recombinant Forkhead-DBD, (Figure 5B lanes 1–4). Thus, the bio-informatically identified sequences were indeed true E4BP4 and XFD1 binding sites. Having determined the specificity of the probes we further tested additional putative E4BP4 binding sites (probes a-d, spanning sites E4 and E5, E6, E7, respectively in Figure 4) that lie within the cluster of sites located at −31 kb. All four new probes bound recombinant E4BP4-DBD, though with different affinities (Figure 5C). We conclude that both E4BP4 and XFD1 sites in intron 1, and the cluster of three sites at −31 kb of *appb* bind zebrafish E4BP4 and Forkhead DBDs efficiently and specifically *in vitro*.

### E4BP4 binding sites in the human APP gene

**Figure 6:**
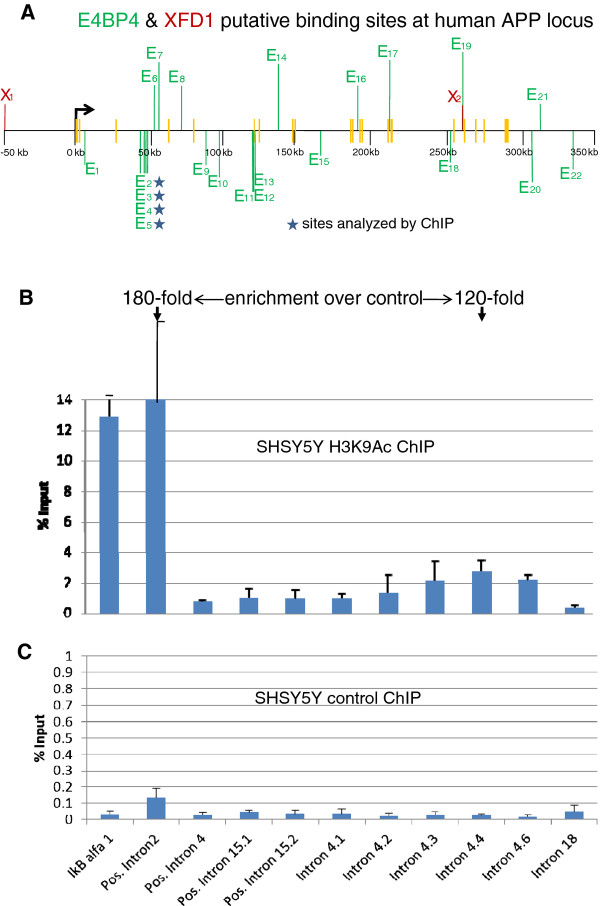
**Panel A: Location of E4BP4 (E, green) and XFD1 (X, red) sites in non-coding DNA within the human APP gene and surrounding 50 kb DNA.** The green and red vertical lines above or below the horizontal line indicate sites in the forward and reverse strand of DNA, respectively. The short yellow vertical bars indicate exons of APP. The first three exons are very close to one another near the transcription start site. Stars indicate the E4BP4 sites marked by H3K9Ac in chromatin immunoprecipitation (ChIP) assays in a human cell-line SHSY5Y expressing APP gene. The bent arrow indicates transcription start site of APP gene. The SHSY5Y cells were analyzed by ChIP using anti-H3K9Ac antibodies as described in Methods. Y-axis represents the amount of material present in anti-H3K9Ac (**Panel B**) or non-specific IgG (**Panel C**) immunoprecipitates compared to input chromatin used for the assay. Note the different scales for the two panels. Results shown are the average of three independent ChIP experiments, each of which was assayed in duplicates. Error bars represent standard deviation between experiments. Amplification from IkBα served as a positive control. The fold enrichment, over control IgG, of H3K9Ac activity at the APP intron 2 site (positive control) and the intron 4 sites are 180-fold versus 120-fold, respectively, as indicated at the top of Panel 6B.

Sequence comparisons of non-coding DNA within and flanking the APP gene in humans and zebrafish did not reveal appreciable conservation at the nucleotide level. However, it was possible that the regulatory logic was evolutionarily conserved between these distantly related species. To determine if the regulatory information obtained in zebrafish had implications for human APP gene regulation, we first scored for E4BP4 binding sites in and around the human APP gene. We noted a cluster of four putative E4BP4 binding sites in intron 4 of human APP (Figure 6A). Locations of these sites are also indicated in the bottom panel of Additional file 5: Figure S5. As a first step towards determining whether these sites were functionally important, we determined the histone modification state around this cluster in a human neuroblastoma cell line, SHSY5Y, which expresses human APP mRNA. Expression of E4BP4 mRNA was also confirmed in the cell line using RT-PCR. We used antibodies directed against histone H3 acetylated at lysine 9 (H3K9Ac) to carry out chromatin immunoprecipitation (ChIP) assays; the co-precipitated genomic DNA was queried by quantitative PCR using primers that identified different parts of the human APP gene (shown in Additional file 2: Figure S2). We detected a modest peak of H3K9Ac activity centered over the region with clustered E4BP4 sites (Figure 6B). As a positive control we used ChIP primers located within the promoter of the ubiquitously expressed IkappaBalpha (IkBα) gene. We also assayed H3K9Ac at a region in intron 2 of human APP that has been previously shown to be enriched for H3K27Ac (http://genome.ucsc.edu/cgi-bin/hgTracks?position=chr21:27252862-27543138&hgsid=203637875&wgEncodeHaibTfbs.Peaks.vis=full), which is another activation-associated histone modification. This region was highly modified with H3K9Ac. The fold enrichment of H3K9Ac at this site and the intron 4 sites was comparable (180-fold versus 120-fold, respectively, (Figure 6B). We conclude that the E4BP4 site-containing intron 4 region is epigenetically modified in these cells.

## Discussion

Several previous reports have described elegant vector systems and procedures to trap enhancers in the zebrafish [27-31]. The methods usually either insert the trap directly into the chromosomes of the organism or test sequences pre-selected based on cross-species conservation, for tissue-specific enhancer activity in Tol2 vectors. Our approach using enhancer-trap BACs is not affected by genome accessibility issues of the enhancer-trap because insertion of the trap occurs in isolated pieces of chromosomal DNA in BACs in the bacterial host (advantages discussed in [32]). Our approach is also likely to be free of biases, as there is no prior selection of sequences to test for enhancer activity. The ability to analyze multiple discontinuous DNA domains that act in concert to regulate expression of a gene such as *appb* appears a likely advantage of the enhancer-trap BAC approach.

A large number of enhancer-trap BACs with deletions/ mutations in the intron 1 enhancer and the upstream −31 kb region were analyzed in zebrafish, and putative binding sites for E4BP4 and XFD1 were identified as being critical for *appb* expression in neurons (Figures 2 and 3, Table 1). The two zebrafish transcription factor proteins, expressed in *E. coli*, bound efficiently and selectively *in vitro* to their respective sites identified as being essential for *appb* expression through mutational analysis (Figure 5). These results were then used to explore whether a similar set of transcription factors could also regulate expression of the human APP gene. We noted that binding sites of E4BP4 and XFD1 were also statistically over-represented at the human APP gene locus (Figure 6A). Chromatin immunoprecipitation (ChIP) analyses with H3K9Ac antibody of a cluster of four E4BP4 sites in intron 4 of human APP indicated that they were epigenetically modified (Figure 6B). It suggests the sites are functionally important in actively transcribing chromatin and are highly likely to serve a regulatory role. SiRNA knock-down experiments to further demonstrate that E4BP4 protein is actually involved in this regulation will need to wait till protocols for efficient transfection of the SHSY5Y neuroblastoma cells are devised.

### Cluster of three E4BP4 sites at −31 kb required for expression of zebrafish *appb*

Expression of the BAC DNAs shown in lanes 20 and 21 of Figure 3B is not in neurons, quite unlike the other clones from the same deletion series. Sequence analyses of the new BAC end created by the lox511-iTol2kan transposon insertion indicated that the cluster of three E4BP4 sites at −31 kb was deleted in both these clones. The dependence of *appb* expression in zebrafish neurons on upstream sequences had also been mapped to that same region in our previous study with deletions made by the enhancer-trap transposon from the opposite loxP end of BAC [14]. The conclusion that ~28 kb of upstream sequence is required for neuronal expression was derived from the strikingly different patterns of expression of the two enhancer-trap *appb* BACs Δ75C and Δ72C (see Figure 7 in reference [14]), which had enhancer-trap locations at ~28 and >31 kb upstream, respectively, of the *appb* transcription start site. That the three E4BP4 sites at −31 kb are important for neuron-specific expression of *appb* in zebrafish is thus re-confirmed here more directly. The three E4BP4 sites at −31 kb also bind E4BP4-DBD efficiently and specifically *in vitro* (Figure 5).

### Some members of Forkhead gene family expressed exclusively in zebrafish notochord

An earlier study [33] indicates the Forkhead family of transcription factors fkd1, fkd2 and fkd4 are expressed during gastrulation in the zebrafish, with high levels of fkd1 and fkd4 mRNA accumulating exclusively in the notochord during somitogenesis. It suggests that fkd1, fkd2 and fkd4 proteins are available only in the notochord and thus could explain the expression of EGFP exclusively in the notochord when the cluster of three upstream E4BP4 sites at −31 kb are deleted in the enhancer-trap *appb*-BACs (Figures 3, 4).

### E4BP4 has repressor activity in addition to activation properties, is intricately involved with the immune system and its expression is clock-regulated

The transcription factor E4BP4, also known as NFIL3, has been known to have both transcription activation and repression activities [34,35]. It serves in the Central Nervous System (CNS) as an anti-apoptotic factor to promote survival and growth of motor neurons [36]. As NFIL3, it is also intricately linked with the immune system, where it is required for protecting natural killer (NK) T cells [37] and regulates IL-12 p40 in macrophages [38]. Strikingly, E4BP4/ NFIL3 has recently been found to regulate the IL12b gene by acting as a repressor from a distal enhancer 10 kb upstream of the gene using the STAT3 pathway [39]. We believe these characteristics of E4BP4/ NFIL3 are very relevant to our findings because the importance of immunological and inflammatory processes in the pathogenesis and therapy of Alzheimer's disease is well documented [40,41].

Results presented here indicate that the cluster of three E4BP4 sites at −31 kb in the zebrafish gene (Figure 4) is critical for neuron-specific expression of EGFP from promoter elements of *appb* in conjunction with the intron enhancer. Earlier reports of E4BP4 having transcription repression activity [34-38], thus facilitates formulating the following working hypothesis for *appb* gene regulation: binding of both E4BP4 and Forkhead proteins to *appb* intron 1 DNA is required for *appb* gene expression. The dependence of neuronal *appb* expression on the three E4BP4 sites at −31 kb could then be explained as follows: with excess E4BP4, possibly from those bound to sequences at −31 kb, Forkhead activity is suppressed, and expression is specific to neurons. In the absence of these upstream sequences, sequestered levels of E4BP4 are low, and expression is in notochord.

### Possible model for *appb* gene regulation

We propose a novel interplay between Fkd and E4BP4/ NFIL3, either directly or indirectly through other proteins, for restricting expression of *appb* to neurons. Although required, the E4BP4 bound to the lone site in the minimal intron 1 enhancer appears not to have enough repressive function to prevent expression in the notochord. Thus in the absence of the cluster of three E4BP4 sites at −31 kb, expression is exclusive to the notochord because that is where fkd1, fkd2 and fkd4 proteins are localized [33]. When the cluster of three E4BP4 sites is present, Forkhead bound to XFD1 is suppressed by E4BP4 proteins and expression is exclusive to neurons. The DNA region 28–31 kb upstream of *appb* is likely to have additional activator sites that enhance neuron-specific expression.

For E4BP4/ NFIL3 and Forkhead to regulate *appb* gene expression in the CNS, the proteins need to be available in the zebrafish brain. Although evidence for availability of these proteins in brain is lacking for zebrafish, expression has been reported for the Forkhead protein in neurons of the mouse spinal cord [42], while the E4BP4/ NFIL3 protein has been shown to be expressed in the embryonic motor neurons of both rat and chicken [36].

### Testing hypothesis for regulation of APP in humans

It is likely that APP gene regulation shares common features in zebrafish and humans. However, the lack of conservation in sequence of non-coding DNA around the APP gene in these model vertebrates has been a dilemma [14]. We explored the hypothesis that conservation may be at the level of the transcription factors involved. A search for E4BP4 and XFD1 sites in and around the APP gene reveals a much greater than statistical frequency of both these sites, just as in the case of the zebrafish *appb* gene. There are 22 putative binding sites for the human E4BP4/ NFIL3, about 6-fold above statistical frequency, as shown in Figure 6A, with an additional one in exon 23 (not shown). Although there are only two sites with the XFD1 consensus sequence, sites with 8 of 9 bases identical (consecutively) to the consensus site exist far more abundantly in human APP (13 additional such sites were identified, but not shown in Figure 6A), leading one to speculate that protein complexes capable of binding to these sites might have evolved to accommodate the single end-nucleotide change. The ChIP experiments using H3K9Ac antibodies to immunoprecipitate actively transcribing chromosomal regions in the undifferentiated SHSY5Y human cell line identifies the cluster of four E4BP4 binding sites in intron 4 as active compared to three other regions in the same gene used as negative controls (shown in Figure 6 panels B and C). These negative controls are introns 15.1, 15.2 and 18 within the same APP gene. It appears likely therefore that E4BP4/ NFIL3 also regulates human APP.

### Variation in levels of β-amyloid in mice brains follows a circadian pattern

The 42-amino acid β-amyloid peptide levels in brain interstitial fluid of mice have been reported to correlate directly with wakefulness [43]. Although the study did not find a similar correlation of full length APP in total tissue homogenates, it is intriguing that expression of the transcription factor E4BP4 shown here to regulate *appb*/ APP expression follows circadian rhythm controls [35].

### Extrapolating results from zebrafish to the human to formulate hypothesis

Identifying gene regulatory DNA domains with conserved function but without conserved sequence across species is a daunting task, especially when they are located differently in the gene region as noted here between *appb* and APP. We propose that regulation of the APP gene in humans occurs by a mechanism similar to that of the *appb* gene in zebrafish, using a similar set of transcription factors that bind to sites distributed differently across the gene in the two species. The zebrafish system allowed rapid identification of important gene-regulatory sequences through mutational analyses. The system also helped delineate between several candidate transcription factor proteins that could potentially bind to the same DNA sequence. A database search for proteins that contain the conserved DNA-binding domain of the Forkhead gene family of transcription factors identified several other DNA-binding proteins. Our ability to focus on the Forkhead family of proteins arose from the previous finding in zebrafish that members of the family fkd1, fkd2 and fkd4 are expressed during gastrulation, with high levels of fkd1 and fkd4 mRNA accumulating exclusively in the notochord during somitogenesis [33].

## Conclusion

Here we have functionally analyzed mutations in the two discontinuous DNA domains in zebrafish *appb* that were shown earlier to be important for expression of the gene in neurons. Previously known transcription factor E4BP4/ NFIL3 and Forkhead binding sites are shown to be required for intron 1 enhancer function. Dependence of neuron specific expression on sequences 31 kb upstream of *appb* is shown to reside in a cluster of three E4BP4 sites. Both E4BP4/ NFIL3 and Forkhead sites in these regulatory domains bind the corresponding zebrafish proteins efficiently and selectively *in vitro.* These sites exist in non-coding DNA of both the zebrafish and human APP genes at levels much above statistical frequency. Furthermore, a cluster of four E4BP4 sites in intron 4 of human APP is shown to be epigenetically marked with H3K9Ac in a human neuroblastoma cell-line that expresses APP. Taken together these findings suggest that *appb* in zebrafish and APP in humans may follow the same regulatory logic using the same set of transcription factors despite a lack of sequence similarity in their regulatory DNA. It suggests potential human APP gene regulatory pathways, not on the basis of comparing DNA primary sequences with zebrafish *appb* but on the model of conservation of transcription factors.

## Abbreviations

APP: Amyloid Precursor Protein (human)/; *appb*: Amyloid Precursor Protein gene (zebrafish)/; TSS: Transcription Start Site/; FIGE: Field Inversion Gel Electrophoresis/; EMSA: Electrophoretic Mobility Shift Assay/; ChIP: Chromatin Immunoprecipitate/; DBD: DNA Binding Domain/; CNS: Central Nervous System; FBS: Fetal Bovine serum.

## Competing interest

The authors declare that they have no competing interests.

## Authors’ contributions

LAS screened enhancer-trap BAC libraries by FIGE, sequenced BAC ends, injected BAC DNA into zebrafish embryos, and documented positive embryos using photo-microscopy. HD helped with construction of the Fkd expression plasmid, purified the proteins, performed EMSA assays with E4BP4 and Fkd proteins and analyzed data. HMW discovered the unique distribution of E4BP4 and XFD1 binding sites in non-coding DNA in and around the zebrafish *appb* and human APP genes, designed and constructed the expression plasmids for E4BP4 and Fkd proteins, and helped write the manuscript. CH screened BAC libraries using FIGE, supplied zebrafish eggs and provided animal care for the zebrafish embryos. DCN supervised zebrafish animal care, analyzed data and helped write the manuscript. PKC designed the study along with RS, helped with design and construction of the enhancer-trap transposon plasmids, generated the enhancer-trap libraries of BAC deletions, purified BAC DNA for injections using Qiagen columns, and was responsible for writing the article with RS. Both RS and PKC critically evaluated the study. PP conducted the ChIP experiments and analyzed data. All authors read and approved the final manuscript.

## Supplementary Material

Additional file 1 **Figure S1.** The *E. coli* expressed DNA-binding protein domain common to members within gene family, shown in green (A) E4BP4/ NFIL3, or red (B) Fkd. The corresponding DNA sequence that was amplified using the PCR primers is underlined. Click here for file

Additional file 2 **Figure S2.** Sequences of primers, analyzed using pDRWN32 software, used to probe chromatin immune-precipitates with H3K9Ac or control IgG antibody. Click here for file

Additional file 3 **Figure S3.** Sequences of primers used to detect E4BP4/ NFIL3 mRNA levels in the human cell line SHSY5Y using RT-PCR. Click here for file

Additional file 4 **Figure S4.** Sequence of intron 1 enhancer is indicated in black letters, and the sequences of predicted transcription factor (TF) binding sites indicated in colored letters. The long arrows indicate the location and directionality of PCR primers used to delete specific transcription factor binding sites. The thick short underlines within the SOX5 site indicate point mutations introduced that leave the overlapping E4BP4 site intact. Click here for file

Additional 5 **Figure S5. ** Locations of putative binding sites of E4BP4 and XFD1 in zebrafish *appb *(from Figure 4) and human APP (from Figure 6A) are tabulated in the top and bottom panels respectively. Click here for file
