# Supplementary material for: Using BAC transgenesis in zebrafish to identify regulatory sequences of the amyloid precursor protein gene in humans
Source: BMC Genomics. 2012 Sep 4;13:451. doi: 10.1186/1471-2164-13-451 (PMC3546842; doi:10.1186/1471-2164-13-451)
Supplement: Additional file 1 — Figure S1. The E. coli expressed DNA-binding protein domain common to members within gene family, shown in green (A) E4BP4/ NFIL3, or red (B) Fkd. The corresponding DNA sequence that was amplified using the PCR primers is underlined. [file 1471-2164-13-451-S1.pdf]

Sequences corresponding to peptide domains common to all members within gene family

A <u>Partial Exon 2 sequence of zebrafish E4BP4 gene - 147 aa E4BP4 peptide synthesized shown in green</u>

GCAAGCCATTAAGAAAGAGCCCCCATGCAGTGGGCCCTACGGTGGAGA<u>GGACGCCTTGGTGCTGGCTGTAG</u>CTTTGCAAG
GGACAGACAGGGACCTGATTAACCACAAACTGTCCACCCTACCTTTCAAGTCCAAGTCCACCAGCTGTCGCCGGAAACGTG
AGTTCATACCCGATGAGAAGAAAGACAACCTATATTGGGAGAGAAGGCGCAAGAACAATGAAGCAGCCAAGCGCTCCAGGG
AAAAGCGCAGACTAAATGACATGGTTCTGGAGAACAAGCTTATGGCCCTTGGGGAAGAGAACGCATCGCTGAAAGCTGAGC
TTTTGTCACTTAAACTCAGGTTTGGTCTAGTGAGCTCAGCCGCTTATGCTCAGGAGGTGCAGAACATCTCCACTTCCACTGC
TGCGCTCTACCAGGACTTTATGTCACCCAGTGCCACCAAGGATTCCTACCCAAGTGATCTGG<u>AGCCAACGCGTTTGACCAGC
AGCTG</u>CATTTCGGTCATCAAGCACTCACCTCACAGCGC

dal vlavalqgtd rdlinhklst lpfkskstsc rrkrefipde kkdnlywerr rknneaakrs rekrrlndmv lenklmalge enaslkaell slklrfglvs saayaqevqn iststaalyq dfmspsatkd sypsdleptr ltss

B <u>Partial Exon 3 sequence of zebrafish FKD1 gene - 154 aa FKD peptide synthesized shown in blue</u>

CCTGCCACCTGCAAACCCTCCAAAG<u>GCATATGCTGAGATCAACTCC</u>AGCGAACCAGAGTTTCAAGAGCTGAAATCAGTTTA
CCGGCGGACATTAAGCCATGCCAAACCGCCGTACTCCTACATCTCTCTCATCTGCATGGCCATCCAGCAGTCTCCAGCCAA
GAGACTGACTCTGAACGAGATCTACGACTGGATCCGTCAGCTCTTCCCATATTACAGACAGAATCAGCAGAGATGGCAGAA
CTCCATCCGCCATTCCCTGTCCTTCAACGACTGCTTCGTGCGTGTCCCGAGATCTCCAGACTCTCCCGGAAAAGGCTCAT
ACTGGGCTTTACACCCTGACTCCGGTAACATGTTTGAAAACGGATGCTACATGCGCCGCCAGAAGCGCTTCAAGTGTCAA
AAATCCACATCAACAGGCAAAAACTCAGAAGGAGAAGACGTGAAAGTGGAAAAGAAGAAAAG<u>GAAGGAGATCAAGTCAGT
CAGCT</u>CCTGTAAATCACCTACAAGTGATGCTACAAAACAG

ayaein ssepefqelk svyrrtlsha kppysyisli cmaiqqspak rltlneiydw irqlfpyyrq nqqrwqnsir hslsfndcfv rvprspdspg kgsywalhpd sgnmfengcy mrrqkrfkcq kststgknse gedvkvekkk rkeiksvs

Note: Both peptides have additional amino acids from vector pET-30a(+)
