# Supplementary material for: Using BAC transgenesis in zebrafish to identify regulatory sequences of the amyloid precursor protein gene in humans
Source: BMC Genomics. 2012 Sep 4;13:451. doi: 10.1186/1471-2164-13-451 (PMC3546842; doi:10.1186/1471-2164-13-451)
Supplement: Additional file 2 — Figure S2. Sequences of primers, analyzed using pDRWN32 software, used to probe chromatin immune-precipitates with H3K9Ac or control IgG antibody. [file 1471-2164-13-451-S2.pdf]

Primer positions in the attached sequence, analyzed in pDRWN32 :

positive intron 2 fp (CAG TCC TTC GCT CTT GAA TCG TTG C)50966
positive intron 2 rp (GAG GAA GGT GAA ACG AGG CGG AGA)51076
positive intron 4 fp (CTC TGT TCT CAG ACA GTA ATA TCT)81034
positive intron 4 rp (GAG AAC AAT CAC ACT GGT AGA G) 81156
positive intron 15-1 fp (CAA GAC TTC TGT GTC CAC CTT GGC) 257181
positive intron 15-1 rp (AAT GCC AGG AGT TGA GCT GA) 257312
positive intron 15-2 fp (GAC CAG CCT ATC CAA CAT AGT GA) 258106
positive intron 15-2 rp (GTG CAC CTC CAC CTC AAC TCA CTG) 258247
intron 4 forward 1 (CCT TCG TGT GAA TGA CCA TCT ATT CC) 90965
intron-4 reverse 1 (GTT TTC CTG AGA TGT TAT ATT GAA ACG T) 91062
intron 4 forward 2 (GAC CCT GTT CAT AGT TGC AGA TAC A) 93592
intron 4 reverse 2 (TCA TAA TGA ACA ACT TAG ACA AAC GGA G) 93759
intron-4 forward 3 (CAA CAG CAG TAA AGT CAG AAG CCA G) 94899
intron-4 reverse 3 (GGG TAT TGC ACT GTG TGG GAA AGA) 95053
intron-4 forward 4 (GCA CAT TCG AAG TCT CAC TGC TCA CA)96572
intron-4 reverse 4 (CTG AAC TCC TTT GTA ATT CAG GCA AC) 96647
intron-4 forward 6 (CAG CGG GTG GTT CAA ATG CAG AAA) 104335
intron-4 reverse 6 (ACA GGG TCT CAA CTA GGA GGG AAA) 104490
intron 18 forward (CAT GTG TGG GCA CCT CAA CCA AAT) 276059
intron 18 reverse (TTA CAG GGC CAT ACA AAC GAG CTG) 276173
