# Supplementary material for: Using BAC transgenesis in zebrafish to identify regulatory sequences of the amyloid precursor protein gene in humans
Source: BMC Genomics. 2012 Sep 4;13:451. doi: 10.1186/1471-2164-13-451 (PMC3546842; doi:10.1186/1471-2164-13-451)
Supplement: Additional file 3 — Figure S3. Sequences of primers used to detect E4BP4/ NFIL3 mRNA levels in the human cell line SHSY5Y using RT-PCR. [file 1471-2164-13-451-S3.pdf]

Primers used for detecting mRNA levels of E4BP4 in the Undifferentiated cell line SHSY5Y

Exon 2,3 fp (GCG CTG GAG GGC GCG GCC T)
Exon 2,3 rp (TCT TCT CCG TCT TAA TCT TCA C)

Exon 3,4 rp (CAC CAC TAG AGT GTT GAC ATG)
or exon 3 fp (GCT GCA GCC TAG TGA AAT C) pairs with
exon 3,4 rp (GAA GAG CTG GCT CCA ATC ATT GT)

exon 4,5 fp (ACT TGT TGG TTC TGT TTA TCA ACT A)
exon 4,5 rp (GCA ATA CTG CAG GAT GCC TTC CTT G)
