# Supplementary material for: Using BAC transgenesis in zebrafish to identify regulatory sequences of the amyloid precursor protein gene in humans
Source: BMC Genomics. 2012 Sep 4;13:451. doi: 10.1186/1471-2164-13-451 (PMC3546842; doi:10.1186/1471-2164-13-451)
Supplement: Additional file 4 — Figure S4. Sequence of intron 1 enhancer is indicated in black letters, and the sequences of predicted transcription factor (TF) binding sites indicated in colored letters. The long arrows indicate the location and directionality of PCR primers used to delete specific transcription factor binding sites. The thick short underlines within the SOX5 site indicate point mutations introduced that leave the overlapping E4BP4 site intact. [file 1471-2164-13-451-S4.pdf]

Intron-1 Enhancer Sequence of *appb* gene

TCCAAAGCATTCTTCTGAGAATCTCTCTCTCTCTCTCTCTCTCTCTCTCTCTCTCTCTATATATA
TATAAAACTAGACCACAGCACAATCCCTTTCGGTCAGTATTGTTATTACATAAGACATCAGC
AATAAAGAAAAAGATTGACCCACTGTAATTGGTTTTCACATTAGTATTGATTCACGTAACAC
TACAGATGAATGGAATCAAGTTTGTGGGTCTTGGTACATGTGTGCTATAGCTTTGCCATTG
TTCCAAAACTGTATTTAGTTAATTTAATTCTTCAAAATTAATTAGTAAGTCTTAAGCATGTCAA
GTGCTTAAATCTGACCTCTTGTAAGTCATTATTTTCTTTCTGACTGGTTCAATAAAAAAGCC
CCTTTAGGCTTTTTTCATTATAGGTGCCACTTGTAGGTACTGTGCCAAAACAATCTTTGACA
GTTTGCGCTGTCACCTCTTTACTGGTCACTATAACCACACACAAGTCATTTTGGCTGAAAG
GTCCTTTGCTTTAGCTTGCTTGTAATTACTAAATCTGTTTTCAGTCAGCCTCCATCTGAAAA
AGGCCTTTCTTTGTTTGACTGGGAAATGTCACTGGAACATGTAAACACGGTCGGTTTTAAA
TTATGTTTTTGAGAGCTACAAAAGCTACTTCATTGATTTTGACAGCCTTCAAGATTAAGCCA
GAGCTGAATGACTCTTGAAAGCTGCACATTTCATTTTGATAGATGCATTAATGTGGATCCT
GAGCTGCTATGACAACCTGACTGAGACACTATTTCGGGAGATTGATATTCGCAGATGATTA
GCTTTCACCAATGGCTCAGTGGATGTCGGTGTTAAAGCATTTTGAGCACCATGGTAATTCT
ATCATAAATGTACATCTATAGCTTATGTGCAGCATACAGAATGATCTTACTACTGCAATGAGC
TGTCGCACACATGGGTTTCCTAAAGAGCTAACTACTGTACTGCGGTCAGTGTAATTACAGT
CTTCTGTTTTTAAACTGCCATACAGCAGAGAGATGTTTTATTGCGCTTT

CTCTCT……ATATAT	**CT-repeat**	CATGTAAAC	**XFD1**
GTATTGTTAT	**SOX5**	ACCATGGTAAT	**OCT1**
TTACATAA	**E4BP4**	GATCTTACT	**GATA3**
