# Supplementary material for: Using BAC transgenesis in zebrafish to identify regulatory sequences of the amyloid precursor protein gene in humans
Source: BMC Genomics. 2012 Sep 4;13:451. doi: 10.1186/1471-2164-13-451 (PMC3546842; doi:10.1186/1471-2164-13-451)
Supplement: Additional 5 — Figure S5. Locations of putative binding sites of E4BP4 and XFD1 in zebrafish appb (from Figure 4) and human APP (from Figure 6A) are tabulated in the top and bottom panels respectively. [file 1471-2164-13-451-S5.pdf]

Locations of E4BP4 & XFD1 putative binding sites at zebrafish *appb* locus

Site	X_1	E_1	X_2	E_2	E_3	E_4	E_5	E_6	E_7	E_8	E_9	E_{10}
Location (kb)	-47.5	-44.5	-44.0	-42.7	-38.7	-38.1	-33.7	-31.6	-31.3	-25.3	-9.3	-3.8

Site	E_{11}	X_3	E_{12}	E_{13}	X_4	X_5	X_6	E_{14}	E_{15}	E_{16}	E_{17}
Location (kb)	2.9	3.3	7.2	10.8	17.6	43.8	47.0	48.3	61.4	69.5	72.2

Locations of E4BP4 & XFD1 putative binding sites at human APP locus

Site	X_1	E_1	E_2	E_3	E_4	E_5	E_6	E_7	E_8	E_9	E_{10}	E_{11}
Location (kb)	-48.6	5.0	42.0	44.7	46.0	47.6	51.5	55.3	72.3	86.5	97.2	120.1

Site	E_{12}	E_{13}	E_{14}	E_{15}	E_{16}	E_{17}	E_{18}	X_2	E_{19}	E_{20}	E_{21}	E_{22}
Location (kb)	118.2	118.3	136.5	164.6	189.0	211.5	251.6	260.6	260.7	307.3	313.4	335.5
